# Brachyury promotes extracellular matrix synthesis through transcriptional regulation of Smad3 in nucleus pulposus

**DOI:** 10.1152/ajpcell.00475.2023

**Published:** 2024-03-04

**Authors:** Yanzhang Xia, Yinghui Wu, Yuhao Gong, Caichun Yue, Linfeng Tao, Tianwen Xin, Cong Shen, Yue Zhu, Minghong Shen, Jun Shen

**Affiliations:** ^1^Department of Orthopedics Surgery, The Affiliated Suzhou Hospital of Nanjing Medical University, Suzhou Municipal Hospital, Gusu School, Nanjing Medical University, Suzhou, People’s Republic of China; ^2^Suzhou Key Laboratory of Orthopedic Medical Engineering, Suzhou University, Suzhou, People’s Republic of China; ^3^Department of Critical Care Medicine, The Affiliated Suzhou Hospital of Nanjing Medical University, Suzhou Municipal Hospital, Gusu School, Nanjing Medical University, Suzhou, People’s Republic of China; ^4^State Key Laboratory of Reproductive Medicine, Center for Reproduction and Genetics, The Affiliated Suzhou Hospital of Nanjing Medical University, Suzhou Municipal Hospital, Gusu School, Nanjing Medical University, Suzhou, People’s Republic of China; ^5^Department of Breast and Thyroid Surgery, The Affiliated Suzhou Hospital of Nanjing Medical University, Suzhou Municipal Hospital, Gusu School, Nanjing Medical University, Suzhou, People’s Republic of China; ^6^Department of Pathology, The Affiliated Suzhou Hospital of Nanjing Medical University, Suzhou Municipal Hospital, Gusu School, Nanjing Medical University, Suzhou, People’s Republic of China

**Keywords:** brachyury, extracellular matrix, intervertebral disc degeneration, nucleus pulposus, Smad3

## Abstract

Metabolic dysfunction of the extracellular matrix (ECM) is one of the primary causes of intervertebral disc degeneration (IVDD). Previous studies have demonstrated that the transcription factor Brachyury* (Bry)* has the potential to promote the synthesis of collagen II and aggrecan, while the specific mechanism is still unknown. In this study, we used a lipopolysaccharide (LPS)-induced model of nucleus pulposus cell (NPC) degeneration and a rat acupuncture IVDD model to elucidate the precise mechanism through which *Bry* affects collagen II and aggrecan synthesis in vitro and in vivo. First, we confirmed Bry expression decreased in degenerated human nucleus pulposus (NP) cells (NPCs). Knockdown of *Bry* exacerbated the decrease in collagen II and aggrecan expression in the lipopolysaccharide (LPS)-induced NPCs degeneration in vitro model. Bioinformatic analysis indicated that *Smad3* may participate in the regulatory pathway of ECM synthesis regulated by Bry. Chromatin immunoprecipitation followed by quantitative polymerase chain reaction (ChIP-qPCR) and luciferase reporter gene assays demonstrated that *Bry* enhances the transcription of *Smad3* by interacting with a specific motif on the promoter region. In addition, Western blot and reverse transcription-qPCR assays demonstrated that *Smad3* positively regulates the expression of aggrecan and collagen II in NPCs. The following rescue experiments revealed that *Bry*-mediated regulation of ECM synthesis is partially dependent on Smad3 phosphorylation. Finally, the findings from the in vivo rat acupuncture-induced IVDD model were consistent with those obtained from in vitro assays. In conclusion, this study reveals that *Bry* positively regulates the synthesis of collagen II and aggrecan in NP through transcriptional activation of Smad3.

**NEW & NOTEWORTHY** Mechanically, in the nucleus, *Bry* enhances the transcription of *Smad3*, leading to increased expression of Smad3 protein levels; in the cytoplasm, elevated substrate levels further lead to an increase in the phosphorylation of Smad3, thereby regulating collagen II and aggrecan expression. Further in vivo experiments provide additional evidence that *Bry* can alleviate IVDD through this mechanism.

## Graphical abstract

**Figure:**
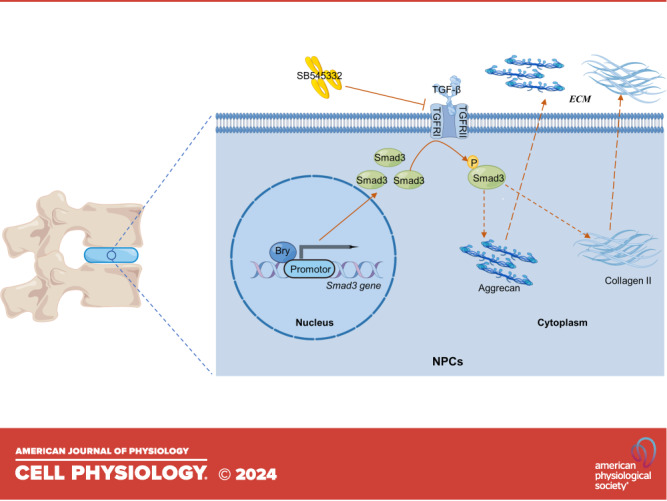

## INTRODUCTION

Intervertebral disk degeneration (IVDD) is widely recognized as the primary cause of low back pain, leading to disability and imposing a significant economic burden on society (1). Current management strategies for IVDD in clinical practice primarily revolve around conservative and surgical treatments (2, 3), which mainly address symptom relief rather than halting the progression of degeneration. The lack of thorough understanding regarding the pathogenesis mechanisms hampers the occurrence of novel therapeutic modalities. IVDD represents an irreversible process characterized by a detrimental cycle of mechanical overloading, degradation of the extracellular matrix (ECM), inflammatory response, and cellular depletion (4–6).

Anatomically, the gel-like nucleus pulposus (NP) is located at the center of the intervertebral disk (IVD), which is encircled by a tough annulus fibrosus and separated from the vertebrae by cartilaginous endplates. Functionally, the gelatinous NP tissue exerts crucial roles in withstanding axial compressional load and providing flexibility for the spinal column (7). The proteoglycan and water in the NP are held together by a fine network of collagen II and other ECM components (8). The metabolic dysfunction of the ECM is one vital cause of the occurrence and progression of IVDD (9). Thus, restoring the balance between the anabolic and catabolic processes of ECM has continuously been the research hotspot in IVD and NP regeneration.

Brachyury (*Bry*), a crucial transcription factor, plays an essential role in preserving the fate of the posterior mesoderm and facilitating the formation of the spine during embryonic development (10). Embryos with mutations impeding *Bry* gene function exhibit defects in notochord differentiation (11). In postnatal NP tissue, *Bry* and several of its target genes, such as *FGF8* (fibroblast growth factor 8), Axin2, pleiotrophin, and *CTGF* (connective tissue growth factor), play crucial roles in NP physiology and are associated with disk degeneration (12–14). A recent study conducted by Tang et al. (15) demonstrated that the sorting of *Bry*-positive human-induced pluripotent stem cells significantly improved the efficiency of differentiation towards NPCs. The above-mentioned evidence suggests that *Bry* is highly involved in NP development and maintenance. Furthermore, Tang et al. (16) reported that the transfection of *Bry* into degenerated human NP cells (NPCs) enhanced the expression of collagen II and aggrecan. Nevertheless, the potential mechanism underlying Bry’s modulation of ECM synthesis remains unclear.

Smad proteins, acting as signal transducers, facilitate the transmission of signals from cell surface receptors to the nucleus (17). A study by Furumatsu et al. (18) reported that Smad2/3 forms transcriptional complexes with SOX9 (SRY-box transcription factor 9) on the enhancer region of collagen II, resulting in the upregulation of collagen II expression in chondrocytes. Another study conducted by Ahn et al. (19) demonstrated that phosphorylated Smad2/3 interacts with the promoter regions of collagen II and aggrecan, leading to an upregulation of the synthesis in chondrocytes. Li et al. (20) reported a significant reduction in the expression of collagen II and aggrecan was observed in the IVD of *Smad3* gene knockout mice. *Smad3* is a vital component and plays important roles in the *TGF-β1/Smads* signaling pathway. Although the impact of *Smad3* on ECM synthesis has been extensively studied in recent decades, the identification of factors governing *Smad3* expression has recently gained prominence as a crucial aspect for unraveling the intricate mechanisms underlying ECM metabolism.

Our preliminary bioinformatics analysis has revealed that *Smad3* is a downstream direct target gene of *Bry*. We hypothesized that *Bry* might promote ECM (collagen II and aggrecan) synthesis by modulating the transcriptional regulation of *Smad3*.

The current study utilized lipopolysaccharide (LPS)-induced NPC degeneration model and rat acupuncture IVDD model to investigate the impact of *Bry* on collagen II and aggrecan synthesis in vitro and in vivo. *Smad3* was confirmed to be the downstream direct target gene of *Bry*. Our research shows that *Bry* can mitigate IVDD via transcriptional activation and phosphorylation of Smad3 to promote collagen II and aggrecan synthesis.

## MATERIALS AND METHODS

### Ethics Statement

All surgical interventions, treatments, and postoperative animal care procedures strictly adhered to the guidelines for Animal Care and Use established by the Committee of Nanjing Medical University (Nanjing, China). Ethical approval was obtained with the reference number IACUC-2004020. The Ethics Committee of the Affiliated Suzhou Hospital of Nanjing Medical University approved the collection of human NP tissue and all experiments involving human NP. These procedures were conducted in accordance with the guidelines established by the Helsinki Declaration (ethical number: KL901339).

### Reagents and Antibodies

LPS was sourced from Sigma-Aldrich (St. Louis, MO). TGF-βR inhibitor was synthesized by Selleck (SB525334, S1476). Additional information regarding the antibodies used in this study can be found in Supplemental Table S1.

### Histological and Immunofluorescence Analyses of Human NP Tissues

Human nucleus pulposus (NP) tissues with Pfirrmann grade II (*n* = 3) were obtained from patients undergoing anterior decompression and fixation operation for thoracic or lumbar burst fractures. NP tissues with Pfirrmann grade IV (*n* = 3) were collected from operated patients diagnosed with lumbar degenerative disease. NP tissues were fixed in 10% formalin for 48 h and then embedded in paraffin wax. Paraffin blocks were sliced into 5-μm sections, which were subsequently stained with hematoxylin and eosin (H&E) and safranin-O/fast green to facilitate histological examination. Costaining of Bry with carbonic anhydrase-12 (CA-12), a marker for NPCs (13, 21), was performed. Briefly, following dewaxing and gradient hydration, the sections underwent antigen retrieval with proteases. Subsequently, the sections were blocked in 1% bovine serum albumin (BSA) for 2 h and incubated overnight at 4°C with primary antibodies against Bry (1:200) and CA-12 (1:200). After rinsing three times, the sections were incubated with the appropriate Alexa Fluor-conjugated secondary antibodies, and the nuclei were counterstained with DAPI (Beyotime, Shanghai, China). Finally, the images were obtained using a Zeiss laser confocal microscope (LSM 810; Carl Zeiss, Oberkochen, Germany).

### Isolation and Culture of NPCs

Male Sprague–Dawley (SD) rats (8-wk-old) were sacrificed by intraperitoneal injection with an excess of sodium pentobarbital (50 mg/kg). Under aseptic conditions, NP tissues were carefully separated from the annulus fibrosus and collected from the caudal disks (Co1–Co5). Then, NP tissues were placed in a sterile centrifuge tube and incubated with 0.5% (wt/vol) type II collagenase for 2 h at 37°C. The isolated NPCs were cultured in the Dulbecco’s modified Eagle’s medium/Nutrient Mixture F-12 (DMEM/F-12; Invitrogen, Waltham, MA) supplemented with 12% fetal bovine serum (FBS; Invitrogen) and 1% streptomycin-penicillin in an incubator with 5% CO_2_ at 37°C. The culture medium was replaced every 2 days, and the second or third passage of NPCs was used for the following experiments.

### Quantitative Real-Time Polymerase Chain Reaction

Total RNA was extracted from NPCs using TRIzol reagent (Invitrogen, Waltham, MA) according to the manufacturer’s instructions. RNA samples (1 μg) were reverse-transcribed into cDNAs with a Prime Script Reverse Transcription Kit (Vazyme, Nanjing, China). qRT-PCR was performed using an ABI 7500 Real-Time PCR system (Applied Biosystems, Foster City, CA). The cycle threshold (CT) values were normalized to the level of 18sRNA. The primer sequences are shown in Supplemental Table S2.

### Western Blot Assay

NPCs were harvested and lysed on ice using radioimmunoprecipitation assay (RIPA) supplemented with 1 mM phenylmethanesulfonyl fluoride (PMSF). Protein concentrations were measured by a bicinchoninic acid (BCA) kit (Beyotime, Shanghai, China). Equivalent amounts of protein were separated on 10% sodium dodecyl sulfate polyacrylamide gel electrophoresis (SDS-PAGE) and blotted onto polyvinylidene difluoride (PVDF) membranes. Then, membranes were blocked in 5% skimmed milk for 1 h and subsequently incubated with primary antibodies (Supplemental Table S1) overnight at 4°C. After rinsing three times, membranes were then incubated with corresponding secondary antibodies. Protein levels were visualized by enhanced chemiluminescence (ECL) and quantified using ImageJ software.

### Cell Transfection

Lentivirus for encoding the brachyury gene was purchased from GenePharma (Shanghai, China). When reaching 30%–40% confluence, NPCs were transduced with lentiviruses at a multiplicity of infection of 30. After 12 h of transduction, the culture medium was replaced with fresh medium. Cells were harvested for further analysis at 72 h post transfection.

All siRNAs were synthesized by GenePharma, and sequences are shown in Supplemental Table S3. The transient transfection of siRNAs was performed using Lipofectamine RNAi MAX (Invitrogen) according to the manufacturer’s instructions. Before further experiments, the transfection efficacy of siRNAs was measured by Western blot analysis.

### Immunofluorescence Staining

After 72 h post treatments, NPCs were fixed with 4% (wt/vol) paraformaldehyde and permeabilized with 0.1% Triton X-100 for 15 min at room temperature. After being blocked with 2% bovine serum albumin (BSA) (wt/vol) (Sigma) for 1 h, specimens were incubated overnight with the respective primary antibodies (Supplemental Table S1) at 4°C. After being incubated with Alexa Fluor-conjugated secondary antibodies (1:1,000) for 1 h and counterstained with DAPI (Beyotime) for 5 min, immunofluorescence images were captured using a confocal laser microscope (Zeiss LSM800; Carl Zeiss, Oberkochen, Germany).

### Luciferase Reporter Assay

293T cells were cultured in 12-well plates and transfected with 0.2 μg of the specific pGL6 luciferase reporter plasmid along with the plasmid encoding the Bry protein. The internal control reporter plasmid containing Renilla luciferase (pRL-TK) was cotransfected with the cells to normalize the transfection efficiency. Two days post transfection, luciferase activity was measured using the Dual-Luciferase Reporter Gene Assay System (Beyotime).

### Chromatin Immunoprecipitation

Chromatin immunoprecipitation (ChIP) assays were conducted according to the established protocol using the EZ-ChIP Kit (Millipore, Billerica, MA). Briefly, NPCs were harvested and subjected to triple washes before cross linking with 1% formaldehyde. Subsequently, the cell lysates were sonicated using a Branson Sonicator 250 to generate 500-bp fragments. The chromatin DNA-protein complex was incubated with Bry antibodies for immunoprecipitation. The input control consisted of approximately 10% of the initial material. The immunoprecipitated DNA was subsequently analyzed by RT-qPCR. The primer sequences are provided in Supplemental Table S4.

### Operation Procedures and Groups

Seventy male Sprague–Dawley rats weighing 400 ± 20 g and aged 12 wk were obtained, and they were randomly divided into two groups of 35 rats each. One group was analyzed 3 days after the operation, and the other group was analyzed 7 days post operation. The rats were housed in a ventilated environment with a temperature of 21°C and an alternating 12-h light-dark cycle. Following 12 h of fasting and 4 h of water deprivation, rats were subjected to anesthesia via intraperitoneal administration of 0.3% pentobarbital. An IVDD model was established using a standard operation procedure as described previously (22). Briefly, a 20-gauge needle was used to puncture the area between the eighth and ninth coccygeal vertebrae (Co8–Co9). To ensure degeneration induction, the needle was rotated for 5 s and held steady for 30 s. Each group of 35 rats was further randomly divided into five subgroups: the Ctr group, IVDD group, LV-NC group, LV-Bry group, and LV-shBry group, with seven rats per subgroup. The Ctr group served as the control group without any treatment. IVDD group: Co8–9 was punctured and injected with 2 μL of PBS. LV-NC group: Co8–9 was punctured and injected with 2 μL lentivirus containing negative control sequences. LV-Bry group: Co8–9 was punctured and injected with 2 μL of lentivirus encoding Bry protein. LV-shBry group: Co8–9 was punctured and injected with 2 μL of lentivirus encoding short hairpin RNA targeting Bry.

### Radiographic Evaluation

At both 3 and 7 days following the surgical procedure, rats in each group (*n* = 7) underwent MRI and X-ray imaging prior to euthanasia. The rats were positioned in a supine position with their tails aligned in a straight line on a mammography device (GE Healthcare, Chicago, IL). Radiographs of the rats were obtained using a collimator-to-film distance of 66 cm, an exposure of 63 mAs, and a penetrating voltage of 35 kV. A sagittal T2-weighted MRI was obtained using a 1.5-T system (GE; fill time, 3000 ms; echo time, 80 ms; field of view, 200 mm × 200 mm; scan thickness, 1.4 mm).

### Histological Analysis

Following radiological examination, the rat caudal Co8–Co9 disks were collected and fixed in 4% paraformaldehyde for 48 h. The disks were then decalcified in a 10% solution of ethylenediaminetetraacetic acid (EDTA) for 45 days before being paraffin-embedded. Sections were cut with thickness of 5 μm and stained with hematoxylin and eosin (H&E) and safranin-O/fast green. The histological scores were evaluated based on criteria previously described by Masuda (23).

### Statistical Analysis

Statistical analyses were conducted using the GraphPad Prism 8 software. All quantitative data were analyzed using either a one-way ANOVA or a two-tailed Student’s *t* test, with the results presented as the means ± standard deviation (SD). Statistical significance was determined at *P* < 0.05, *P* < 0.01, and *P* < 0.001.

## RESULTS

### Bry Expression Decreases in Degenerated Human NPCs

**Figure 1.**
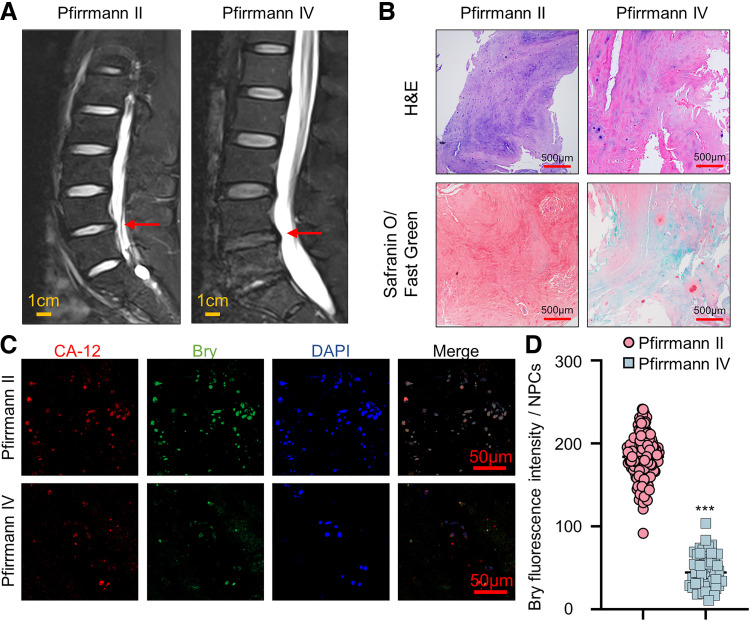
Low expression of Brachyury (Bry) in human degenerative nucleus pulposus (NP) tissue. *A*: T2-weighted MRI scans showing intervertebral discs classified as Pfirrmann grade II and IV (*n* = 3 for each group, red arrows highlight the respective IVD regions). *B*: histological evaluation of human NP tissues using hematoxylin and eosin (H&E) and safranin-O/fast green staining (scale bar = 500 μm). *C*: immunofluorescence analysis of CA-12 and Bry in human NP tissues (scale bar = 50 μm). *D*: quantification of Bry expression in human NP cells. Experiments in (*C*) were performed three times. The data are presented as means ± standard deviation (SD). ***P < 0.001, compared with the Pfirrmann grade II group. CA-12, carbonic anhydrase-12; IVD, intervertebral disc degeneration; MRI, magnetic resonance image.

To investigate the potential involvement of *Bry* in the pathogenesis of IVDD, NP tissues were collected from clinical cases with different degrees of degeneration. Figure 1*A* displays spinal magnetic resonance images (MRIs) of patients with IVDD, which are classified by the Pfirrmann grading system. H&E and safranin-O/fast green results showed a reduction in the cellularity of NPCs and ECM contents in the *grade IV* group relative to the *grade II* group (Fig. 1*B*). Costaining of Bry with CA-12 demonstrated a significant decrease in the cellularity of NPCs and the expression of Bry in the *grade IV* group compared with the *grade II* group (Fig. 1, *C* and *D*). Taken together, these data suggest a low expression of Bry in degenerated NPCs.

### Knockdown of Bry Exacerbates the Reduction of ECM Expression in LPS-Induced NPC Degeneration

**Figure 2.**
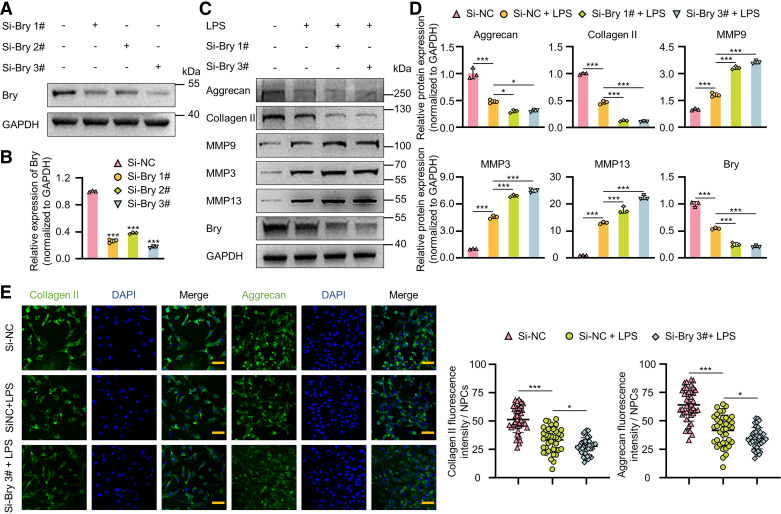
*Bry* knockdown exacerbates the reduction of ECM expression in LPS-induced degeneration of nucleus pulposus cells (NPCs). *A* and *B*: Western blot analysis and quantitative assessment of Bry protein levels in rat NPCs transfected with three distinct siRNAs (50 nM) for 72 h. Rat NPCs were transfected with siRNAs (50 nM) and incubated with 10 ng/mL LPS for 72 h or left untreated. *C* and *D*: Western blot analysis of protein levels of collagen II, aggrecan, MMP9, MMP3, MMP13, and Bry. *E*: immunofluorescence staining to assess the levels of collagen II and aggrecan in rat NPCs following treatment with LPS and siRNA transfection. Scale bar: 100 μm. Experiments in *A*, *C*, and *E* were performed three times. The statistical significance was determined using *P < 0.05, ***P < 0.001. The data are shown as means ± standard deviation (SD). ECM, extracellular matrix; MMP, matrix metalloproteinase.

An LPS-induced NPC degeneration model was utilized to investigate the relationship between *Bry* and ECM expression. Based on our preliminary study, NPC degeneration was induced with 20 μg/mL of LPS for 72 h (24). Three independent siRNAs were used to knock down *Bry* in rat NPCs. Based on the Western blot results, siBry 1# and 3# were chosen for further experiments due to their effective knockdown of *Bry* (Fig. 2, *A* and *B*). Knockdown of *Bry* led to a significant reduction in the expression of aggrecan and collagen II, whereas the expression of MMP9, MMP3, and MMP13 was significantly upregulated (Supplemental Fig. S1*A*). To further explore the impact of *Bry* on NPC degeneration, an LPS-induced NPC degeneration model was utilized for investigation. As indicated in Fig. 2, *C* and *D*, the protein expression of aggrecan and collagen II significantly decreased, whereas MMP9, MMP3, and MMP13 markedly increased with LPS treatment. In LPS-treated NPCs, knocking down *Bry* resulted in a further decrease in ECM expression and a further increase in MMPs if experiments were consistent with the Western blot results (Fig. 2*E*). Furthermore, the knockdown of *Bry* significantly increased the total MMP activity in LPS-treated NPCs (Supplemental Fig. S1*B*). In addition, ELISA analysis targeting aggrecan and collagen II revealed that *Bry* knockdown significantly reduced their levels in the extracellular compartment during LPS-induced NPC degeneration (Supplemental Fig. S1*C*). Taken collectively, LPS-induced NPC degeneration was found to be exacerbated with the knockdown of *Bry*, resulting in a further reduction of ECM expression.

### Potential Roles of Smad3 in Bry-Mediated ECM Synthesis

**Figure 3.**
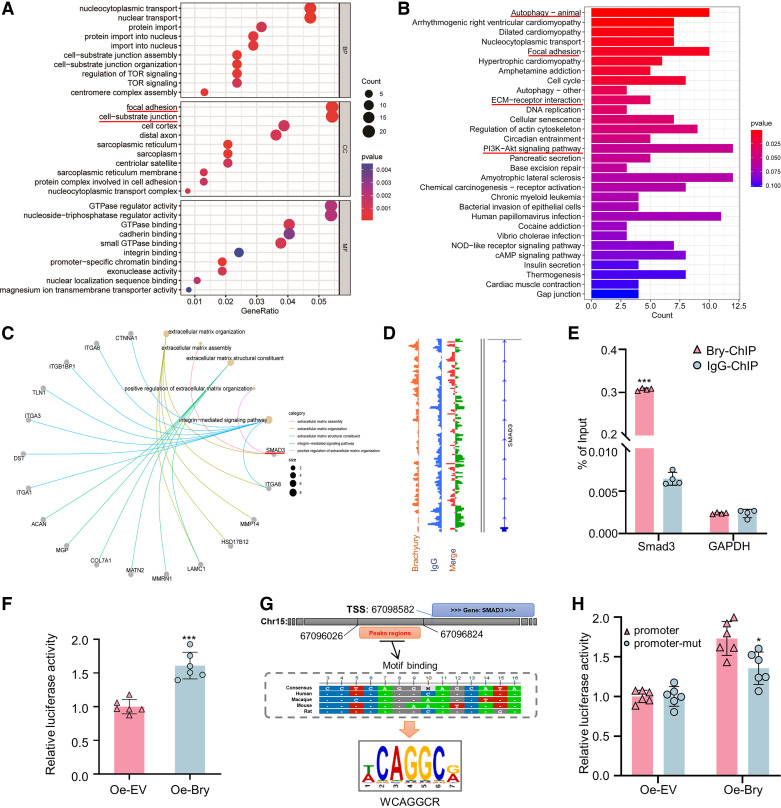
Potential involvement of *Smad3* in *Bry*-mediated extracellular matrix (ECM) synthesis in nucleus pulposus cells (NPCs). *A*: gene ontology (GO) enrichment analysis identified various biological processes (BP), cellular components (CC), and molecular functions (MF). *B*: Kyoto Encyclopedia of Genes and Genomes (KEGG) analysis revealed significant enrichment in key biological pathways. *C*: five significantly enriched GO categories highly associated with ECM synthesis. *D*: localization of Bry-binding peak within the *Smad3* promoter region. *E*: chromatin immunoprecipitation followed by PCR (ChIP-PCR) analysis of *Smad3* in rat NPCs. ChIP was performed using Bry antibody, and PCR amplification was carried out. Input DNA represents total chromatin prior to immunoprecipitation. The *GAPDH* gene served as a negative control. *F*: luciferase activity assays were performed in 293T cells transfected with luciferase reporter constructs and treated with plasmids encoding Bry (1 μg/mL) for 48 h. *G*: sequence analysis of the putative Bry-binding region. *H*: luciferase activity assays were performed in 293T cells transfected with luciferase reporter constructs containing the *Smad3* promoter or mutated promoter and treated with plasmids encoding Bry (1 μg/mL) for 48 h. Experiments in *E*, *F*, and *H* were performed three times. The statistical significance was determined using ***P < 0.001. The data are presented as means ± standard deviation (SD).

To further explore the molecular mechanism contributing to the exacerbated reduction of ECM expression after *Bry* knockdown in LPS-induced NPC degeneration, Bry ChIP-seq data were analyzed. Gene ontology (GO) analysis revealed a significant enrichment of *Bry*-target genes in various cellular components, with notable enrichment observed in the focal adhesion and cell-substrate junction (Fig. 3*A*). KEGG analysis of *Bry*-target genes has shown significant enrichment in several key biological pathways that have been reported to be highly associated with ECM synthesis. These pathways include autophagy, focal adhesion, ECM-receptor interaction, and PI3K-Akt signaling (Fig. 3*B*). As shown in Fig. 3*C*, *Smad3* exhibits significant enrichment in ECM organization, ECM assembly, and positive regulation of ECM organization, highlighting its strong association with ECM metabolism. Therefore, we hypothesized that *Smad3* plays a crucial role in *Bry*-mediated synthesis of ECM. Figure 3*D* demonstrates *Bry*-binding peaks mainly localized on the promoter region of *Smad3*. ChIP-qPCR assays revealed a significant enrichment of Bry in the promoter region of *Smad3* in rat NPCs (Fig. 3*E*).

To further explore the regulatory effect of *Bry* on *Smad3* transcription, pGL6 firefly luciferase reporter vectors were utilized to clone the complete promoter sequences of *Smad3* (Supplemental Fig. S2). *Bry* overexpression significantly augmented the luciferase activity of reporter vector containing *Smad3* promoter sequences in 293T cells (Fig. 3*F*). Moreover, bioinformatic analysis of the genomic region harboring the peak of Bry binding revealed a conserved 13-base pair sequence across human, macaque, mouse, and rat genomes. This conserved sequence encompassed a functional motif referred to as 5′-WCAGGCR-3′ (Fig. 3*G*). Furthermore, a mutant promoter was synthesized that retained the structural characteristics of the *Smad3* promoter sequence but removed the Bry-binding motif. The reporter assay demonstrated that the mutant promoter markedly attenuated the luciferase activity of *Smad3* mediated by Bry (Fig. 3*H*). These data suggest that *Smad3* may play potential roles in *Bry*-mediated ECM synthesis.

### Regulation of Smad3 Expression by Bry in NPCs

**Figure 4.**
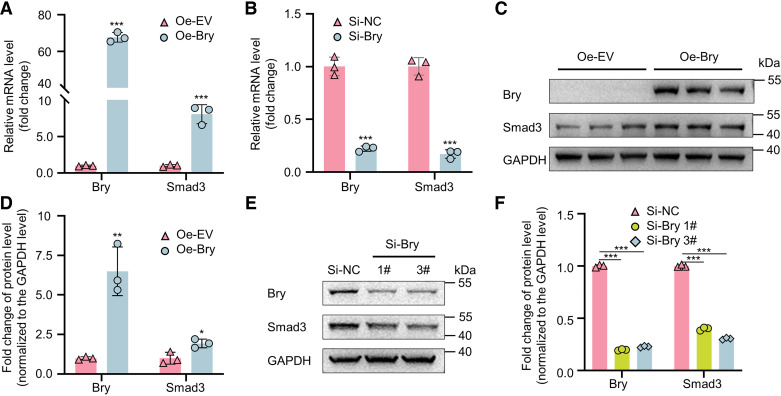
Regulation of *Smad3* expression by *Bry* in NPCs. *A*: RT-qPCR analysis of *Bry* and *Smad3* expression in rat NPCs transfected with lentivirus Bry (MOI = 100) for 72 h. *B*: RT-qPCR analysis of *Bry* and *Smad3* expression in rat NPCs transfected with siBry (50 nM) for 72 h. *C* and *D*: Western blot and quantitative analysis of Bry and Smad3 expression in rat NPCs transfected with lentivirus Bry (MOI = 100) for 72 h. *E* and *F*: Western blot and quantitative analysis of *Bry* and *Smad3* expression in rat NPCs transfected with siBry (50 nM) for 72 h. Experiments in *A*, *B*, *C*, and *E* were performed three times. The statistical significance was determined using *P < 0.05, **P < 0.01, ***P < 0.001 compared with the control group. The data are presented as means ± SD. MOI, multiplicity of infection; NPC, nucleus pulposus cells; RT-qPCR, reverse transcription quantitative polymerase chain reaction.

To investigate the regulatory impact of *Bry* on the expression of *Smad3* in NPCs, we utilized lentivirus expressing Bry or siRNA targeting *Bry* to manipulate Bry expression. RT-qPCR results demonstrated that the upregulation of *Bry* markedly enhanced the expression of *Smad3* (Fig. 4*A*), whereas *Bry* knockdown diminished *Smad3* mRNA expressions (Fig. 4*B*). The results of Western blots were consistent with the RT-qPCR results (Fig. 4, *C*–*F*). Overall, *Bry* positively regulated the *Smad3* transcription in NPCs.

### Smad3 Regulates ECM Synthesis in Rats NPCs in Vitro

**Figure 5.**
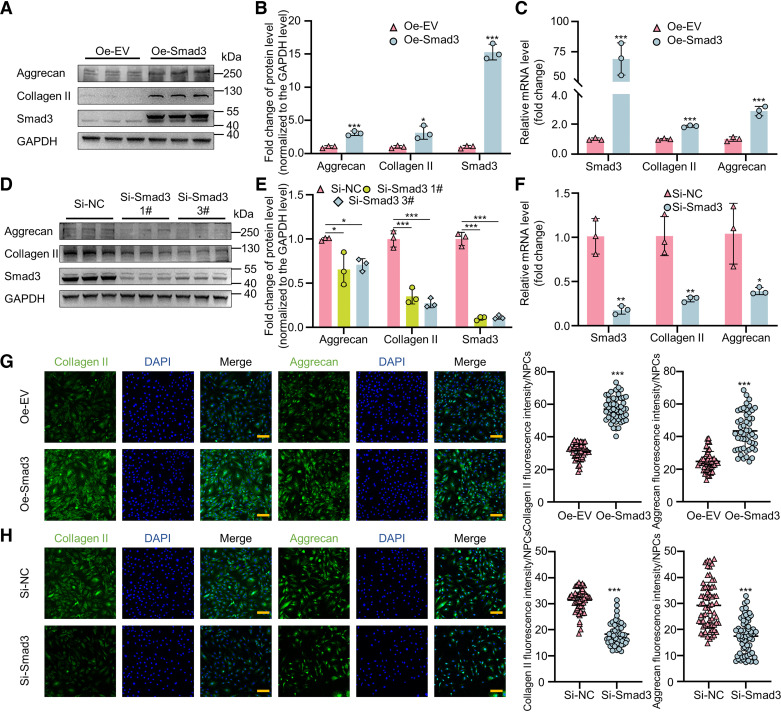
*Smad3* positively regulates ECM synthesis in rats NPCs in vitro. *A* and *B*: Western blot and quantitative analysis of Smad3, collagen II, and aggrecan expression in rat NPCs transfected with plasmids encoding Smad3 (1 μg/mL) for 48 h. *C*: RT-qPCR analysis of Smad3, collagen II, and aggrecan expression in rat NPCs transfected with plasmids encoding Smad3 (1 μg/mL) for 48 h. *D* and *E*: Western blot and quantitative analysis of Smad3, collagen II, and aggrecan expression in rat NPCs transfected with siSmad3 1# and 3# (50 nM) for 48 h. *F*: RT-qPCR analysis of Smad3, collagen II, and aggrecan expression in rat NPCs transfected with siSmad3 1# and 3# (50 nM) for 48 h. *G*: immunofluorescent and quantitative analysis of collagen II and aggrecan in rat NPCs transfected with plasmids encoding Smad3 (1 μg/mL) for 48 h. *H*: immunofluorescent and quantitative analysis of collagen II and aggrecan in rat NPCs transfected with siSmad3 (50 nM) for 48 h. Scale bar: 100 μm. All experiments were performed three times. *P < 0.05; **P < 0.01; ***P < 0.001 compared with the negative control group. The data are shown as means ± SD. ECM, extracellular matrix; NPC, nucleus pulposus cells; RT-qPCR, reverse transcription quantitative polymerase chain reaction.

*Smad3* was overexpressed by transfecting plasmids into rats NPCs to explore the effect on ECM synthesis. The RT-qPCR and Western blotting assay consistently revealed a statistically significant increase in the expression of aggrecan and collagen II at both the mRNA and protein levels following *Smad3* overexpression (Fig. 5, *A*–*C*). Three independent siRNAs 1#, 2#, and 3# were used to knock down the expression of *Smad3*. WB results revealed that siSmad3 1# and 3# show higher knockdown efficiency and were selected for further experiments (Supplemental Fig. S3). *Smad3* knockdown resulted in a notable decrease in the expressions of aggrecan and collagen II at both the mRNA and protein levels (Fig. 5, *D*–*F*). The IF results were consistent with the findings of WB and RT-qPCR assays (Fig. 5, *G* and *H*). These data demonstrate that *Smad3* could positively regulate ECM synthesis in rat NPCs.

### Bry-Mediated Regulation of ECM Synthesis Exhibits Partial Dependency on Smad3 Phosphorylation

**Figure 6.**
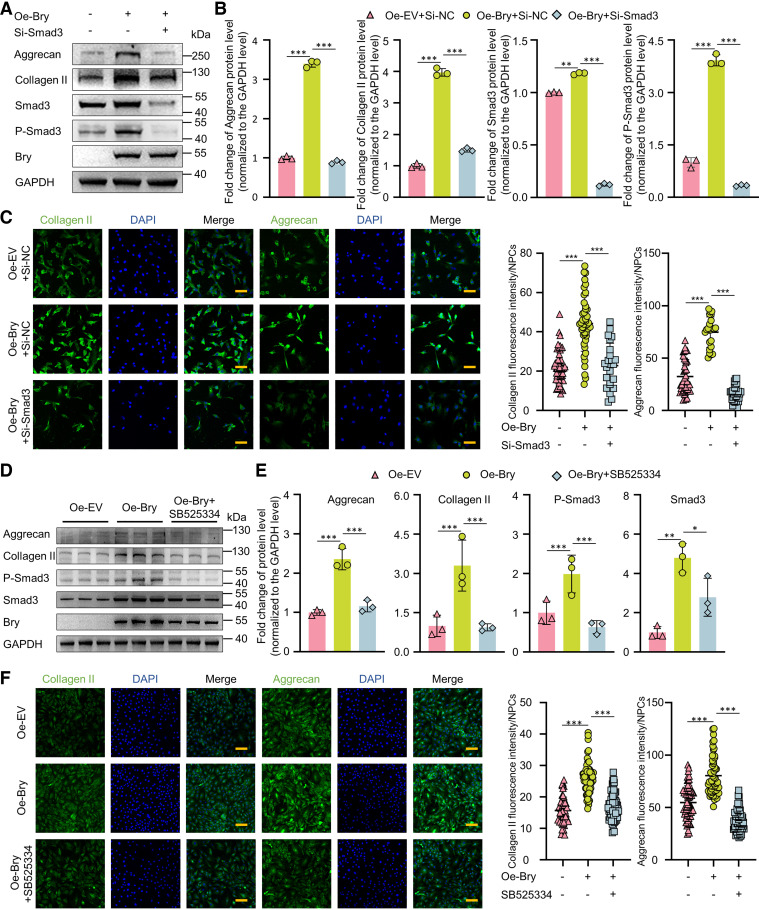
*Bry*-mediated regulation of ECM synthesis partially dependent on Smad3 phosphorylation. *A* and *B*: Western blot analysis and quantitative assessment of Bry, Smad3, P-Smad3, collagen II, and aggrecan protein levels in rat NPCs transfected with lentivirus Bry (MOI = 100) for 24 h and treated with siSmad3 1# (50 nM) for 48 h or left untreated. *C*: immunofluorescent and quantitative analysis of Bry, Smad3, collagen II, and aggrecan protein levels in rat NPCs transfected with lentivirus Bry (MOI = 100) for 24 h and treated with siSmad3 1# (50 nM) for 48 h or left untreated. *D* and *E*: Western blot analysis and quantitative assessment of Bry, Smad3, P-Smad3, collagen II, and aggrecan protein levels in rat NPCs transfected with lentivirus Bry (MOI = 100) and incubated with 5 μM SB525334 for 72 h or left untreated. *F*: immunofluorescent and quantitative analysis of Bry, Smad3, collagen II, and aggrecan protein levels in rat NPCs transfected with lentivirus Bry (MOI = 100) and incubated with 10 μM SB525334 for 72 h or left untreated. Scale bar: 100 μm All experiments were performed three times. *P < 0.05; **P < 0.01; ***P < 0.001 compared with the negative control group. The data are shown as means ± SD. MOI multiplicity of infection; NPC, nucleus pulposus cells.

To further investigate the role of *Smad3* on *Bry*-mediated regulation of ECM synthesis, rescue assays were performed. WB results showed knocking down *Smad3* resulted in a notable reduction of the elevated expression of aggrecan and collagen II by *Bry* overexpression (Fig. 6, *A* and *B*). The IF results were consistent with WB (Fig. 6*C*). The CCK-8 assay results revealed that exposure to 5 μM SB525334, a selective inhibitor of TGF-β receptor I (ALK5), for 72 h did not impact the proliferation of NPCs (Supplemental Fig. S4*A*). Moreover, treatment with 5 μM SB525334 significantly suppressed the phosphorylation of Smad3 (Supplemental Fig. S4*B*). Furthermore, the treatment of SB525334 in *Bry*-overexpression NPCs inhibited the phosphorylation of Smad3 and significantly decreased the elevated expression of aggrecan and collagen II (Fig. 6, *D* and *E*). In addition, the fluorescence intensity of aggrecan and collagen II also showed a significant decrease upon inhibiting the phosphorylation of Smad3 (Fig. 6*F*). Overall, the enhanced synthesis of ECM mediated by *Bry* is partially reliant on the phosphorylation of Smad3.

### Bry Modulates Progression of IVDD In Vivo

**Figure 7.**
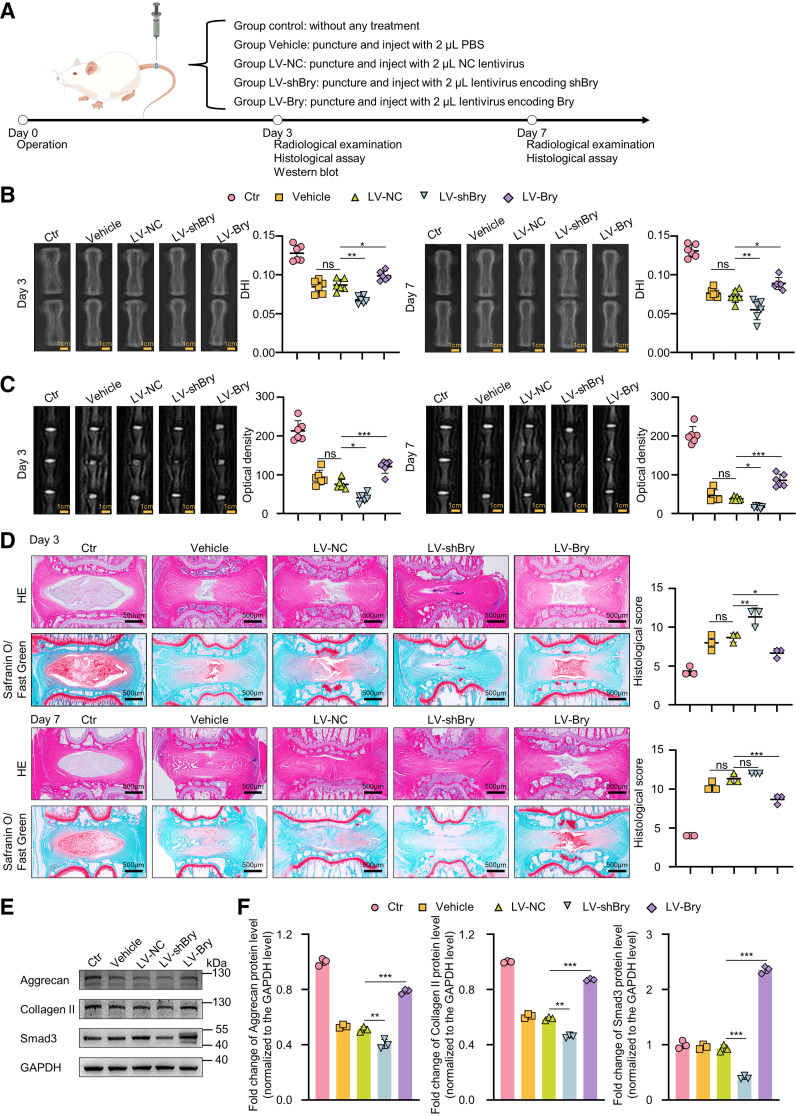
*Bry* modulates progression of IVDD in vivo. *A*: schematic illustration of in vivo experimental regimens. *B*: radiograph of coccygeal vertebrae and changes of DHI at *days 3* and *7* post operation (*n* = 6 for each group). *C*: representative MRI scans of coccygeal vertebrae and optical density of IVD at *days 3* and *7* post operation. *D*: histological staining and histological scores of IVDD model at *days 3* and *7* post operation. Scale bar: 500 μm. *E* and *F*: Western blot analysis and quantitative assessment of Smad3, collagen II, and aggrecan protein levels in rat NP tissues at *day 3* post operation (*n* = 3 for each group). DHI, disk height index; IVDD, intervertebral disc degeneration; MRI, magnetic resonance image; NP, nucleus pulposus.

To investigate the effect of *Bry* on IVDD in vivo, a rat model was established using needle acupuncture (Fig. 7*A*). X-ray results indicated that, at *day 3* post operation, the disk height index (DHI) decreased by 34% and 33% in the Vehicle and LV-NC groups, respectively, compared with the control group. Similarly, at *day 7* post operation, a decrease of 34% and 33% in DHI was observed in the Vehicle and LV-NC groups, respectively, compared with the control group. No significant difference in DHI was found between the Vehicle and LV-NC groups at both *days 3* and *7* post operation. At *day 3* post operation, the LV-shBry group exhibited a 22% decrease in the DHI compared with the LV-NC groups, whereas the LV-Bry group showed a 13% increase. Likewise, at *day 7* post operation, the LV-shBry group exhibited a 24% decrease and the LV-Bry group showed a 23% increase (Fig. 7*B*). Furthermore, MRI scans indicated that IVD optical density decreased by 57% and 65% in the Vehicle and LV-NC groups at *day 3* post operation, and by 87% and 81% at *day 7* post operation compared with the control group. No statistically significant difference was found between the Vehicle and LV-NC groups at both *days 3* and *7* post operation. In comparison with the LV-NC group, the LV-shBry group showed a 46% reduction in IVD optical density at *day 3* post operation and a 57% reduction at *day 7* post operation. Conversely, the LV-Bry group exhibited a 1.6-fold and 2.22-fold increase, respectively, at *day 3* and *day 7* post operation (Fig. 7*C*). H&E and safranin-O/fast green staining revealed that, in the control group, the NP appeared as large, full elliptical shapes. The other four groups exhibited irregular shapes, and their NP volume was smaller than that of the control group. In addition, the LV-shBry group exhibited lower cellularity and decreased proteoglycan content at both *days 3* and *7* post operation compared with the LV-NC group. In contrast, the LV-Bry group exhibited a significant increase in proteoglycan content and cellularity of NPCs, with a more regular microstructure at both *days 3* and *7* post operation compared with the LV-NC group. The histological score in the LV-shBry group increased by 1.3-fold and 1.06-fold, respectively, at *days 3* and *7* post operation, compared with the LV-NC group. Conversely, the score in the LV-Bry group decreased by 25% at *day 3* and by 27% at *day 7* post operation, compared with the LV-NC group (Fig. 7*D*). The WB indicated that, at *day 3* post operation, both the Vehicle and LV-NC groups exhibited decreased expression of aggrecan and collagen II when compared with the control group. However, there was no significant difference in the expression of Smad3. Moreover, in comparison with the LV-NC group, the LV-shBry group showed obviously reduced expression of aggrecan, collagen II, and Smad3, whereas the LV-Bry group exhibited higher expression of aggrecan, collagen II, and Smad3 (Fig. 7, *E* and *F*). These results suggest that *Bry* overexpression alleviates puncture-induced IVDD, whereas *Bry* knockdown exacerbates it.

**Figure 8.**
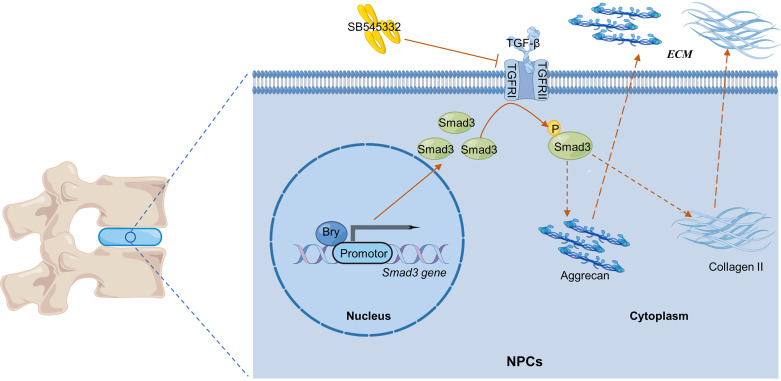
Schematic illustration showing that *Bry* transcriptionally regulates *Smad3* for positive modulation of ECM in NPCs. ECM, extracellular matrix; NPC, nucleus pulposus cells.

Our findings demonstrate that *Bry* positively regulates the synthesis of ECM by promoting *Smad3* transcription in NPCs. In the nucleus, *Bry* enhances the transcription of *Smad3*, leading to increased expression of Smad3 protein levels. In the cytoplasm, elevated substrate levels further lead to increased phosphorylation of Smad3, thereby regulating collagen II and aggrecan expression (Fig. 8).

## DISCUSSION

Investigations into molecular biology-based therapies have shown significant potential in delaying or reversing the progression of IVDD. Several studies have suggested that growth factors such as *GDF5* (growth differentiation factor 5), *TGF* (transforming growth factor), and *PDGF* (platelet-derived growth factor) are instrumental in facilitating the synthesis of ECM in NPCs (25, 26). However, due to their short half-life in cells and rapid degradation and elimination, growth factors exhibit transient effects despite their beneficial properties (27). In contrast, the stable transfection of transcription factors allows for prolonged expression in NPCs, facilitating long-term transcriptional regulation of downstream target genes. Our study reveals that the transcription factor *Bry* can regulate the expression of aggrecan and collagen II, which implies that *Bry* has the potential to serve as a target for the treatment of IVDD.

The *Bry* gene plays a crucial role in mesoderm formation and notochord development (28). *Bry* also has the potential for the biological repair and treatment of IVDD. The study by Tang et al. (15) showed that higher and spatially uniform expression of NP markers and glycosaminoglycans in sorted *Bry*-GFP^+^ cell pellets was observed as compared with sorted *Bry*-GFP^−^ cell pellets. Another study (16) revealed a significant increase in glycosaminoglycan protein levels in degenerated NPCs after 2 wk of overexpressing *Bry* compared with the sham group. These studies have sparked our interest in exploring the possible role of *Bry* in the expression of ECM in NPCs. Our previous research indicates that *Bry* regulates the transcription of aggrecan by binding to a specific motif on the promoter region and promotes ECM synthesis in adult NPCs (24). As depicted in Fig. 6, the knockdown of *Smad3* or inhibition of Smad3 phosphorylation significantly diminished the upregulated expression of aggrecan and collagen II caused by *Bry* overexpression, which provides insight into the underlying mechanism through which *Bry* mediates the regulation of ECM synthesis.

The Smad family includes R-Smads, Co-Smads, and I-Smads. As the main member of R-Smads and a hallmark protein in the *TGF-β/Smads* signaling pathway, *Smad3* has been implicated in diverse biological processes, including energy metabolism (29), embryonic development (30), fibrosis (31), and cancer progression (32). Smad3 and Smad2 are phosphorylated by the TGF-β receptor I, and subsequently phosphorylated Smad2/3 translocates into the nucleus with Smad4, regulating the transcription of target genes (33). Li et al. (34) reported that knocking down *AP-2α* inhibited the expression of *Smad3* while increasing the expression of collagen II and aggrecan in a rat IVDD model, which is contradictory to our results and the observation by Li et al. (20). It is likely that the relationship between *Smad3* and ECM synthesis is regulated by phosphorylation, activation, and other factors.

The relationship between *Bry* and *Smad3* may vary among species or different cell lines. Faial et al. (35) discovered that *Bry* and *Smad2/3* signaling cooperate to regulate the endoderm differentiation in human embryonic stem cells. Dahle et al. (36) found that *Bry* is a direct target of *Smad2/3* signaling in mouse embryonic stem cells, which differs from our findings. Another report suggested that there is a positive feedback loop between the *TGF-β/Smad* signaling pathway and *Bry*, which controls the epithelial-mesenchymal transition in human carcinoma cells (37). These findings suggest that there may be a complex relationship between *Bry* and *Smad3*.

One primary limitation of this study is its failure to determine if *Bry* overexpression might elicit unintended consequences in NPCs or adjacent tissues. Gene overexpression as a therapeutic approach can lead to disrupted cellular pathways and unforeseen physiological reactions, potentially attenuating therapeutic efficacy or engendering harmful side effects (38). In addition, the risk of tumorigenesis associated with the overexpression of growth factors or related genes cannot be overlooked, as these elements typically undergo stringent regulation in normal cellular physiology (37, 39). Regarding Smad3 phosphorylation, although the current research indicates a facilitative function in ECM synthesis, elevated phosphorylation levels of Smad3 may also influence a spectrum of cellular activities. For instance, *Smad3* participates in the *TGF-β* signaling pathway, a critical regulator of cellular proliferation, differentiation, and apoptosis (40, 41). Consequently, augmenting Smad3 phosphorylation may favor ECM synthesis while simultaneously modulating other *Smad3*-mediated mechanisms. The enduring consequences of prolonged Smad3 phosphorylation on NPC vitality and the structural integrity of the NP tissue warrant extensive research. Subsequent investigations should also investigate the interplay between *Bry* and NPC senescence and apoptosis, potentially revealing broader roles for *Bry* and offering strategies to enhance the regenerative process while curtailing adverse outcomes.

The present study has demonstrated that *Bry* positively regulates the synthesis of collagen II and aggrecan in NPCs through transcriptional activation and phosphorylation of Smad3. Furthermore, a specific Bry-binding motif has been identified in the promoter region of *Smad3*.

## DATA AVAILABILITY

The datasets used in the current study are available from the corresponding author on reasonable request.

## SUPPLEMENTAL DATA

10.6084/m9.figshare.25020584 Supplemental Tables S1–S4 and Figs. S1–S4: https://doi.org/10.6084/m9.figshare.25020584.

## GRANTS

This work was supported by Key Project of Social Development in Jiangsu—Clinical Frontier Technology (Grant No. BE2021659, to J.S.); Gusu Health Talent Project of Suzhou (GSWS2020056, to J.S.); and the Technology Project of Suzhou Health Commission (LCZX202210, to J.S.).

## DISCLAIMERS

It is important to note that the funders were not directly involved in the design of the study, the analysis of the data, or the interpretation of the results.

## DISCLOSURES

No conflicts of interest, financial or otherwise, are declared by the authors.

## AUTHOR CONTRIBUTIONS

Y.X., Y.W., and J.S. conceived and designed research; Y.X., Y.G., C.Y., and M.S. performed experiments; Y.X., Y.W., C.Y., L.T., C.S., and Y.Z. analyzed data; Y.W., Y.G., and C.Y. interpreted results of experiments; Y.X. and Y.W. prepared figures; Y.X. and Y.G. drafted manuscript; T.X. and J.S. edited and revised manuscript; J.S. approved final version of manuscript.
